# Mitochondrial DNA Activates the NLRP3 Inflammasome and Predisposes to Type 1 Diabetes in Murine Model

**DOI:** 10.3389/fimmu.2017.00164

**Published:** 2017-02-27

**Authors:** Daniela Carlos, Frederico R. C. Costa, Camila A. Pereira, Fernanda A. Rocha, Juliana N. U. Yaochite, Gabriela G. Oliveira, Fernando S. Carneiro, Rita C. Tostes, Simone G. Ramos, Dario S. Zamboni, Niels O. S. Camara, Bernhard Ryffel, João S. Silva

**Affiliations:** ^1^Department of Biochemistry and Immunology, Ribeirão Preto Medical School, University of São Paulo, Ribeirao Preto, Brazil; ^2^Department of Pharmacology, Ribeirão Preto Medical School, University of São Paulo, Ribeirao Preto, Brazil; ^3^Department of Pathology and Legal Medicine, Ribeirão Preto Medical School, University of São Paulo, Ribeirao Preto, Brazil; ^4^Department of Molecular and Cell Biology, Ribeirão Preto Medical School, University of São Paulo, Ribeirao Preto, Brazil; ^5^Department of Immunology, Institute of Biomedical Sciences (ICB), University of São Paulo, São Paulo, Brazil; ^6^University of Orleans and CNRS, INEM, Molecular Immunology, UMR6218, Orleans, France; ^7^IDM, Institute of Infectious Disease and Molecular Medicine, University of Cape Town, Cape Town, South Africa

**Keywords:** NLRP3 inflammasome, type 1 diabetes, cytokines, IL-17-producing CD4 T cells (Th17), IL-17-producing CD8 T cells (Tc17), IFNγ-producing CD4 T cells (Th1), IFNγ-producing CD8 T cells (Tc1)

## Abstract

Although a correlation between polymorphisms of NOD-like receptor family-pyrin domain containing 3 (NLRP3) and predisposition to type 1 diabetes (T1D) has been identified, the potential function and activation of the NLRP3 inflammasome in T1D have not been clarified. The present study shows that non-obese diabetic mice exhibited increased NLRP3, and pro-IL-1β gene expression in pancreatic lymph nodes (PLNs). Similar increases in gene expression of NLRP3, apoptosis associated speck like protein (ASC) and pro-IL-1β were induced by multiple low doses of streptozotocin (STZ) in C57BL/6 mice. In addition, diabetic C57BL/6 mice also exhibited increased IL-1β protein expression in the pancreatic tissue at day 7, which remained elevated until day 15. Diabetic mice also showed increased positive caspase-1 macrophages in the PLNs, which were decreased in NLRP3^−/−^ mice, but not in ASC^−/−^ mice, after STZ treatment. NLRP3- and IL-1R-deficient mice, but not ASC-deficient mice, showed reduced incidence of diabetes, less insulitis, lower hyperglycemia, and normal insulin levels compared to wild-type (WT) diabetic mice. Notably, these mice also displayed a decrease in IL-17-producing CD4 and CD8 T cells (Th17 and Tc17) and IFN-γ-producing CD4 and CD8 T cells (Th1 and Tc1) in the PLNs. Following STZ treatment to induce T1D, NLRP3-deficient mice also exhibited an increase in myeloid-derived suppressor cell and mast cell numbers in the PLNs along with a significant increase in IL-6, IL-10, and IL-4 expression in the pancreatic tissue. Interestingly, diabetic mice revealed increased circulating expression of genes related to mitochondrial DNA, such as cytochrome *b* and cytochrome *c*, but not NADH dehydrogenase subunit 6 (NADH). Mitochondrial DNA (mDNA) from diabetic mice, but not from non-diabetic mice, induced significant IL-1β production and caspase-1 activation by WT macrophages, which was reduced in NLRP3^−/−^ macrophages. Finally, mDNA administration *in vivo* increased Th17/Tc17/Th1/Tc1 cells in the PLNs and precipitated T1D onset, which was abolished in NLRP3^−/−^ mice. Overall, our results demonstrate that mDNA-mediated NLRP3 activation triggers caspase-1-dependent IL-1β production and contributes to pathogenic cellular responses during the development of STZ-induced T1D.

## Introduction

Type 1 diabetes (T1D) results from the autoimmune destruction of insulin-producing pancreatic β cells (1) in genetically predisposed individuals. It is currently known that both innate and adaptive immune responses play a role in the pathogenesis of the disease. Adaptive immunity has been studied thoroughly over the past few years with several therapies, such as anti-CD3 monoclonal antibody therapy (2), anti-CD20 (3), and antithymocyte globulin treatment, reaching clinical trials (4). However, whether the innate immune response triggers T1D remains poorly understood and controversial. In non-obese diabetic (NOD) mice, a deficiency of toll-like receptor (TLR) 2 (5) or the MyD88 adaptor molecule (6) correlated with protection from developing T1D, yet TLR2^−/−^ (7) and MyD88^−/−^ (8) mice are susceptible to T1D induced by multiple low doses of streptozotocin (MLD-STZ).

Although many studies in the literature on innate immunity focused on the TLRs in T1D, the contribution of nucleotide binding and oligomerization domains (NOD), such as nucleotide-binding domain-like receptor (NLR), in the development of T1D needs to be explored. The NOD-like receptor family-pyrin domain containing 3 (NLRP3) is a member of the NLR family. NLRP3 assembles a complex called the inflammasome through oligomerization with apoptosis-associated speck-like protein (ASC) in myeloid cells, such as dendritic cells (9) and macrophages (10). This process leads to the autocatalytic activation of caspase-1 (11), which in turn cleaves pro-IL-1β and pro-IL-18 into mature forms (12). The NLRP3 inflammasome is thought to play an important role as a defense mechanism against pathogens and damage signals called danger-associated molecular patterns (DAMPs), such as uric acid crystals, ATP, high-mobility group box 1, and the heat-shock proteins hsp70 and hsp90 (13).

Other activators, such as pore-forming toxins (14) and UV radiation, also activate the NLRP3 inflammasome by reducing intracellular K+ concentrations or by promoting cytosolic release of lysosomal cathepsins (13). Certain activators, such as ATP, are able to induce mitochondrial dysfunction and apoptosis, which results in the release of oxidized mitochondrial DNA (mDNA) into the cytosol then binds and activates the NLRP3 inflammasome (12). The role of the NLRP3 inflammasome in autoinflammatory diseases, such as type 2 diabetes (T2D) (15), and autoimmune diseases, such as experimental autoimmune encephalomyelitis (EAE) (16), has been recognized. In this context, inhibition of caspase-1 suppresses IL-17 production by CD4 T cells and γδ T cells and the induction of EAE, which suggests that IL-1β induces the Th17 responses in autoimmunity (17). In fact, IL-1β synergizes with IL-6 and IL-23 to trigger the expression of the IRF4 and RORγt transcription factors as well as driving the induction of Th17 cells (18).

Despite this evidence, little is known about the role of the NLRP3 inflammasome in T1D. Interestingly, an association study in Brazil identified two single-nucleotide polymorphisms in NLRP3 that are associated with T1D (19). A recent study has also demonstrated that NLRP3 deficiency plays a protective role against T1D by inhibiting chemokines and chemokine receptors involved in immune cell migration to pancreatic islets (20). However, the activator of the NLRP3 receptor in T1D and the precise immunological mechanisms related to T1D pathogenesis remain elusive. Our data demonstrate that mDNA isolated from diabetic mice displays an intrinsic capacity to activate the NLRP3 inflammasome in macrophages. Furthermore, increased expression of mDNA-related genes was detected in diabetic mice sera. Finally, mDNA administration causes IL-1β production associated with the induction of pathogenic Th17/Tc17/Th1/Tc1 responses in the pancreatic lymph nodes (PLNs), which results in STZ-induced T1D onset.

## Results

### Diabetic Mice Have Upregulation of NLRP3 Inflammasome Gene Expression and IL-1β Production in PLNs and Pancreas

**Figure 1:**
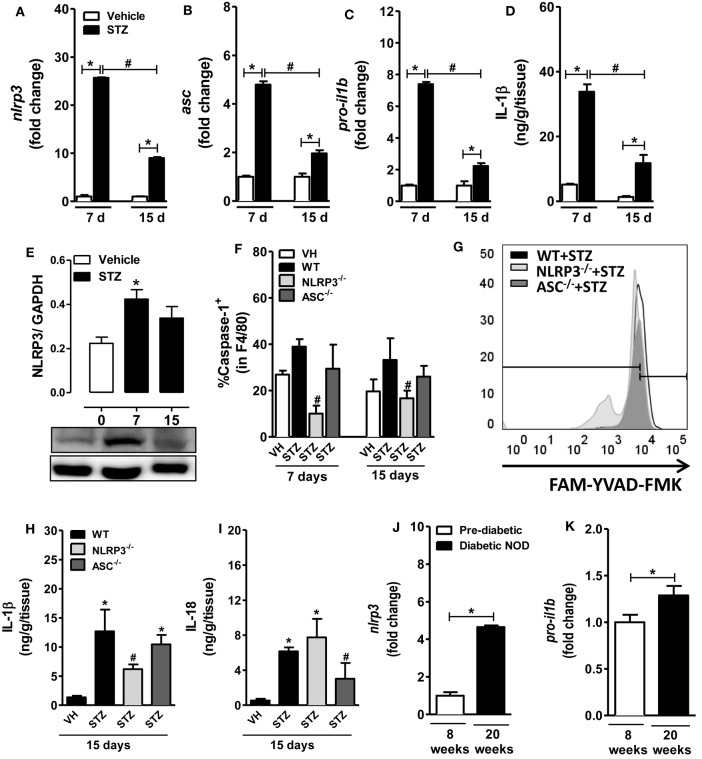
**NOD-like receptor family-pyrin domain containing 3 (NLRP3) inflammasome-related protein and gene expression profile during type 1 diabetes**. Relative gene expression of NLRP3 **(A)**, apoptosis-associated speck-like protein (ASC) **(B)**, and pro-IL-1β **(C)** by RT-PCR in the pancreatic lymph nodes (PLNs) of streptozotocin (STZ)-induced diabetic or non-diabetic mice (vehicle). The time course of IL-1β production was determined in the pancreatic tissue by an ELISA assay **(D)**. The kinetics of NLRP3 expression was quantified 7 or 15 days after STZ by Western blotting **(E)**. The PLNs were removed, and caspase-1 activation was measured with a fluorescent cell-permeable probe that binds to activated caspase-1 (FLICA), after 7 and 15 days of STZ or vehicle administration **(F,G)**. The concentrations of IL-1β **(H)** and IL-18 **(I)** were determined in the pancreatic tissue by an ELISA assay. The relative gene expression of NLRP3 **(J)** and pro-IL-1β **(K)** was assessed by RT-PCR in the PLNs of prediabetic (8 weeks of age) and non-obese diabetic (NOD) mice (20 weeks of age). The results are expressed as the mean ± SEM (*n* = 9 in the vehicle-injected wild-type (WT) group; *n* = 15 in the STZ-administered WT group; *n* = 15 in the STZ-administered ASC^−/−^ group; *n* = 15 in the STZ-administered NLRP3^−/−^ group; *n* = 18 in the prediabetic group and *n* = 12 in the diabetic NOD group). *p ≤ 0.05 compared to the vehicle-injected WT group or prediabetic group, ^#^*p* ≤ 0.05 compared to the STZ-administered WT group. Significant differences between two groups were compared by Student’s *t*-test or three or more groups by one-way ANOVA followed by Tukey’s multiple-comparison test. The results are representative of a single experiment repeated three times.

To investigate the role of the NLRP3 inflammasome in the pathogenesis of T1D, C57BL/6 wild-type (WT) male mice were inoculated intraperitoneally with MLD-STZ (40 mg/kg) for five consecutive days and were assessed for mRNA expression of NLRP3, ASC and pro-IL-β in the PLNs at 7 and 15 days after starting the STZ injections. Increased gene expression of the NLRP3, ASC, and pro-IL-1β genes was found in the PLNs of diabetic mice at 7 and 15 days after STZ treatment compared to non-diabetic mice treated with the vehicle (Figures 1A–C). We also observed a peak of protein IL-1β levels at day 7 after STZ-induced T1D, which decreased at day 15, but remained significantly elevated compared to non-diabetic mice (Figure 1D). In addition, we detected a significant increase of NLRP3 protein expression on day 7 using a Western blot, which remained increased at day 15 after STZ-induced T1D (Figure 1E). In agreement, we noted an increase of caspase-1-positive macrophage percentage at day 7, which slightly decreased at day 15 in the PLNs after STZ-induced T1D (Figure 1F). Notably, we observed a significant decline in the caspase-1-positive macrophage percentage in the PLNs of NLRP3-deficient mice, but not in ASC-deficient mice, not only on day 7 but also on day 15 compared to WT mice after STZ treatment (Figures 1F,G). Similarly, the IL-1β protein levels decreased significantly in the pancreatic tissue of NLRP3-deficient mice, but not in ASC-deficient mice after STZ treatment (Figure 1H). Conversely, the IL-18 protein levels were reduced significantly only in the pancreatic tissue of ASC-deficient mice compared to WT mice after STZ treatment (Figure 1I). Additionally, genetically determined NOD mice displayed increased gene expression of NLRP3 and pro-IL-1β in the PLNs compared to prediabetic mice (Figures 1J,K). These findings demonstrated that NLRP3-dependent IL-1β expression and caspase-1 activation is induced in macrophages in PLNs during T1D.

### IL-1R Signaling Confers Susceptibility to the Development of STZ-Induced T1D

**Figure 2:**
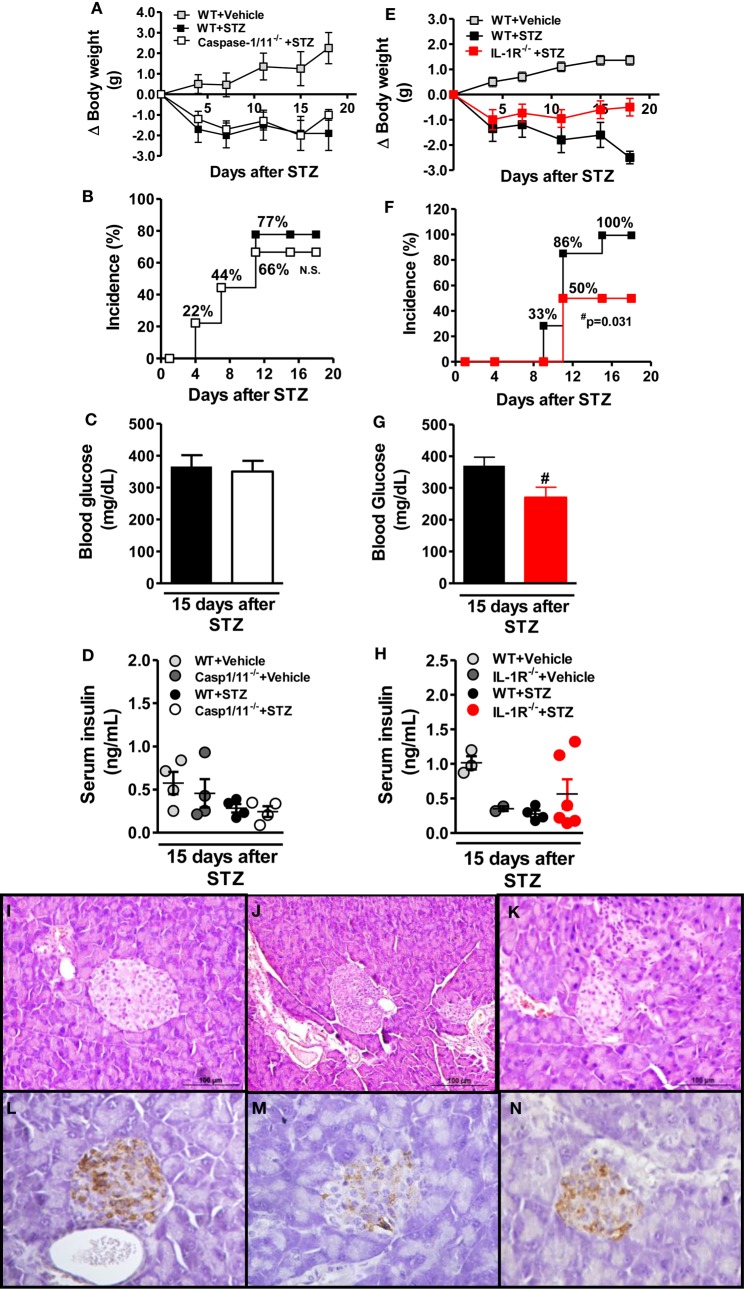
**IL-1R deficiency, but not caspase-1/11, confers resistance to type 1 diabetes development**. Body weight variation **(A,E)**, cumulative disease incidence **(B,F)**, and blood glucose levels **(C,G)** were detected in caspase-1/11^−/−^, IL-1R^−/−^, or wild-type (WT) mice. These clinical parameters were monitored 1, 4, 7, 11, 15, and 18 days after the initial streptozotocin (STZ) treatment. The non-diabetic mice only received the sodium citrate (vehicle). The serum insulin concentrations were determined at day 15 after the initiation of STZ or vehicle administration **(D,H)**. Pancreatic tissues from vehicle-injected WT **(I,L)**, STZ-treated WT **(J,M)**, and STZ-treated IL-1R^−/−^ mice **(K,N)** were stained with hematoxylin-eosin (H&E) (upper panels) or immunostained for insulin (lower panels), respectively (original magnification 400×). The results are expressed as the mean ± SEM (*n* = 12 in the vehicle-injected WT group; *n* = 18 in the STZ-administered WT group; *n* = 18 in the STZ-administered caspase-1/11^−/−^ group; and *n* = 18 in the STZ-administered IL-1R^−/−^ group). ^#^*p* ≤ 0.05 compared to the STZ-administered WT group. Significant differences between the two groups were compared by Student’s *t*-test or three groups by one-way ANOVA followed by Tukey’s multiple-comparison test. The results are representative of a single experiment repeated three times. n.s., not significant.

Diabetic WT mice exhibited body weight loss compared to non-diabetic mice (Figures 2A,E). On the other hand, whereas caspase-1/11^−/−^ mice maintained the body weight loss (Figure 2A), IL-1R^−/−^ mice had a very minor body weight loss after STZ treatment (Figure 2E). Similarly, 66% of caspase-1/11^−/−^ mice in comparison to 50% of IL-1R^−/−^ mice developed T1D at day 15 after STZ treatment (Figures 2B,F). Additionally, blood glucose levels were significantly lower in IL-1R^−/−^ mice (Figure 2G), but were not affected in the caspase-1/11^−/−^ mice compared to WT mice (Figure 2C). Serum insulin levels increased in IL-1R^−/−^ (Figure 2H), but not in caspase-1/11^−/−^ mice (Figure 2D) compared to WT mice after 15 days of the STZ treatment, although the increase in IL-1R^−/−^ animals was not significant. The pancreatic islets from non-diabetic WT mice appeared to be structurally normal with no leukocytic infiltration (Figure 2I). However, pancreatic islets of diabetic WT mice revealed invasive insulitis (Figure 2J), whereas a less extensive inflammatory infiltration was observed in the IL-1R^−/−^ mice after STZ (Figure 2K). Moreover, immunostaining showed the islets of non-diabetic mice had a high number of β cells containing insulin (Figure 2L). The islets of diabetic WT mice had fewer β cells (Figure 2M), while those cells from the IL-1R^−/−^ mice had many more β cells positive for insulin (Figure 2N). Collectively, these results suggest that IL-1R signaling contributes to pancreatic islet inflammation, which leads to insulin-producing β cell damage and development of T1D.

### IL-1R Signaling Increases Pathogenic Th1/Th17/Tc17 Populations during STZ-Induced T1D

Despite the fact that there was no observable difference in the percentage of CD4^+^IL-17^+^ cells (Figure S1A in Supplementary Material), the absolute number of this population was significantly reduced in the PLNs of IL-1R^−/−^ compared to diabetic WT mice after STZ treatment (Figure S1D in Supplementary Material). In addition, the percentage of CD8^+^IL-17^+^ cells did not differ among the several experimental groups (Figure S1B in Supplementary Material), but there was a significant decrease in the absolute number of this cell population in the PLNs of IL-1R^−/−^ mice compared to diabetic WT mice (Figure S1E in Supplementary Material). The frequency and absolute numbers of CD4^+^IFN-γ^+^ cells in the PLNs were significantly reduced in IL-1R^−/−^ mice compared to diabetic WT mice (Figures S1C,F in Supplementary Material). There was a significant increase in the protein IL-6 levels in the pancreatic tissue of IL-1R^−/−^ mice compared to the diabetic WT mice (Figure S1G in Supplementary Material). Despite the lack of differences in expression levels, IL-17 decreased in IL-1R^−/−^ mice compared to diabetic WT mice after STZ treatment (Figure S1H in Supplementary Material). Despite a trend toward increase, no differences in the IFN-γ or TNF-α expression between the experimental groups were observed (Figures S1I,J in Supplementary Material). In contrast, the IL-4 and IL-10 levels were significantly increased in the pancreatic tissues of IL-1R^−/−^ mice compared to diabetic WT mice after STZ treatment (Figures S1K,L in Supplementary Material). These data support the idea that the IL-1R signaling pathway plays an important role in driving the Th17/Tc17/Th1 immune response after STZ induces T1D.

### NLRP3 Activation Is Required for Insulitis and Development of STZ-Induced T1D

**Figure 3:**
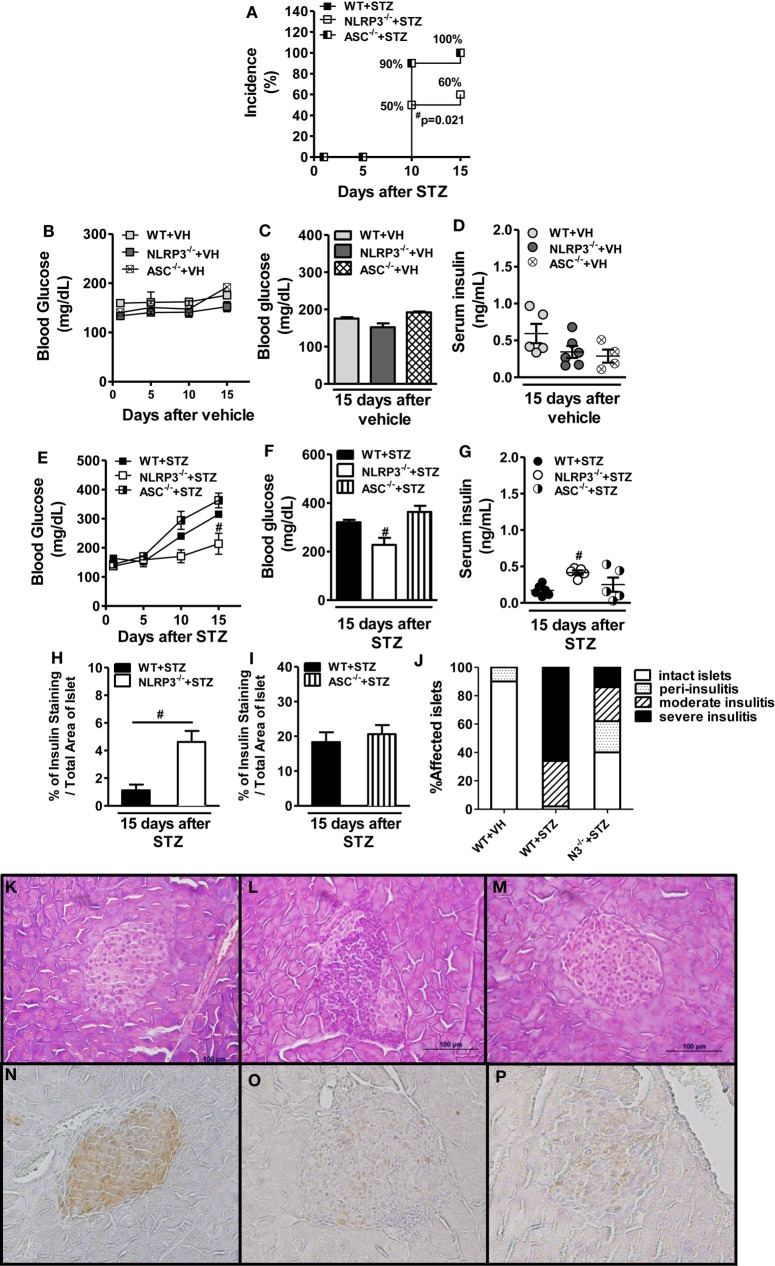
**NOD-like receptor family-pyrin domain containing 3 (NLRP3) deficiency promotes protection against type 1 diabetes development**. Cumulative disease incidence was monitored in NLRP3^−/−^, ASC^−/−^, or wild-type (WT) mice after streptozotocin (STZ) administration **(A)**. The time course of glycemia was monitored or blood glucose levels was determined at day 15 after the initiation of vehicle **(B,C)** or STZ administration **(E,F)** by a glucometer system Accu-Chek Active. The serum insulin concentrations were determined at day 15 after the initiation of STZ or vehicle administration **(D,G)**. The quantitative analysis of insulin-staining pancreatic islets **(H,I)** or semiquantitative scale insulitis score **(J)**. Pancreatic tissues from vehicle-injected WT **(K,N)**, STZ-treated WT **(L,O)**, and STZ-treated NLRP3^−/−^ mice **(M,P)** were stained with hematoxylin-eosin (H&E) (upper panels) or immunostained for insulin (lower panels), respectively (original magnification 400×). The results are expressed as the mean ± SEM (*n* = 12 in the vehicle-injected WT group; *n* = 24 in the STZ-administered WT group; *n* = 12 in the vehicle-injected ASC^−/−^ group; *n* = 24 in the STZ-administered ASC^−/−^ group; *n* = 12 in the vehicle-injected NLRP3^−/−^ group; and *n* = 24 in the STZ-administered NLRP3^−/−^ group). ^#^*p* ≤ 0.05 compared to the STZ-administered WT group. Significant differences between two groups were compared by Student’s *t*-test or three or more groups by one-way ANOVA followed by Tukey’s multiple-comparison test. The results are representative of a single experiment repeated three times or a compilation of two different experiments (A).

To further explore whether NLRP3 and ASC are involved in T1D onset, we used the MLD-STZ model in WT, NLRP3^−/−^, and ASC^−/−^ mice, and disease incidence was monitored. STZ-treated diabetic WT and ASC^−/−^ mice had a cumulative disease incidence of 100%, while the NLRP3^−/−^ mice developed resistance and had a reduction of disease incidence of 40% (Figure 3A). As expected, WT, NLRP3^−/−^, and ASC^−/−^ mice did not become hyperglycemic and had normal serum insulin levels after vehicle administration (Figures 3B–D). The WT and ASC^−/−^ mice became hyperglycemic at day 15 after STZ treatment, whereas NLRP3^−/−^ had a significant decrease of glycemia levels after STZ treatment (Figures 3E,F). Importantly, NLRP3^−/−^ presented higher levels of insulin in the serum, whereas STZ-injected ASC^−/−^ mice maintained unaltered insulin levels compared to WT after STZ administration (Figure 3G). Corroborating this observation, these mice displayed increased insulin immunohistochemistry staining in the pancreatic islets, whereas ASC^−/−^ mice had similar staining to WT mice after STZ treatment (Figures 3H,I).

In STZ-administrated mice, we found the appearance of more invasive insulitis and reduction of insulin-positive β cells (Figures 3J,L,O). Later, we investigate whether the protection observed in NLRP3^−/−^ mice against STZ-induced T1D could be attributed to a reduced pro-inflammatory response into the pancreatic islets. In fact, histological analysis showed that STZ-injected NLRP3^−/−^ mice had milder inflammatory infiltration with less severe insulitis in the pancreatic islets and an increase of insulin-positive β cells (Figures 3J,M,P). Non-diabetic WT mice did not have moderate or severe insulitis and showed intense insulin-positive β cells in the pancreatic islets (Figures 3J,K,N). Taken together, our data indicate that an NLRP3-dependent mechanism is required for pancreatic islet inflammation, which results in insulin-producing β cell damage and T1D development.

### NLRP3 Activation Increases Th17/Tc17 and Decreases the Myeloid-Derived Suppressor Cell (MDSC) Populations during STZ-Induced T1D

**Figure 4:**
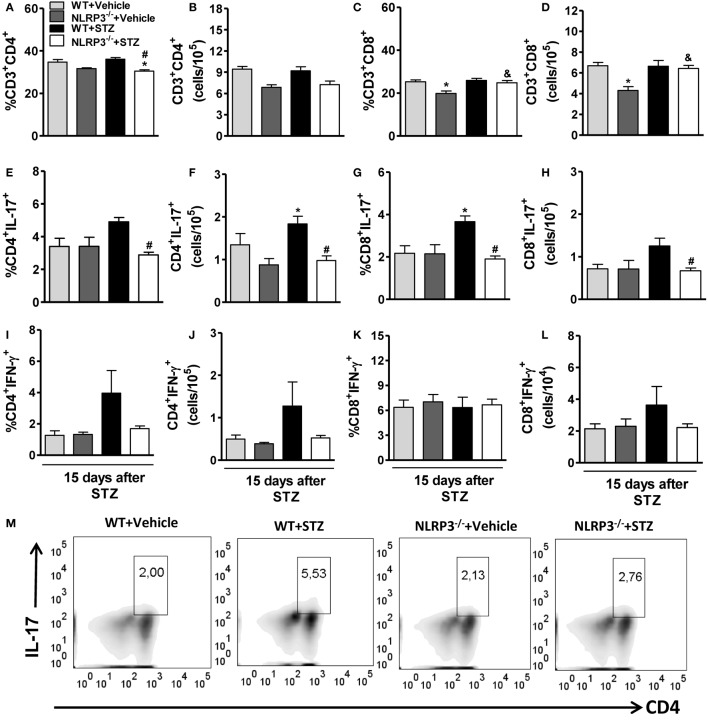
**NOD-like receptor family-pyrin domain containing 3 (NLRP3) deficiency decreases the Th7/Tc17 cell populations in the pancreatic lymph nodes (PLNs) in type 1 diabetes**. PLN cells from vehicle-injected wild-type (WT) (light gray bars), NLRP3^−/−^ (dark gray bars), streptozotocin (STZ)-administered WT mice (black bars), or NLRP3^−/−^ (white bars) mice were harvested 15 days after the initiation of STZ or vehicle administration. The percentage and absolute numbers of CD3^+^CD4^+^ **(A,B)**, CD3^+^CD8^+^ **(C,D)**, CD4^+^IL-17^+^ **(E,F)**, CD8^+^IL-17^+^ **(G,H)**, CD4^+^IFN-γ^+^ **(I,J)**, and CD8^+^IFN-γ^+^ **(K,L)** cells were determined in the PLNs by flow cytometry. Th17 percentages in the PLNs are shown in representative dot plots **(M)**. Intracellular cytokine levels were detected after stimulation with PMA plus ionomycin. The gate was set on CD3-positive lymphocytes. The results are expressed as the mean ± SEM (*n* = 12 in the vehicle-injected WT group; *n* = 24 in the STZ-administered WT group; *n* = 12 in the vehicle-injected NLRP3^−/−^ group; and *n* = 24 in the STZ-administered NLRP3^−/−^ group). *p ≤ 0.05 compared to vehicle-injected WT group, ^#^*p* ≤ 0.05 compared to STZ-administered WT group, or ^&^*p* ≤ 0.05 compared to vehicle-injected NLRP3^−/−^ group. Significant differences between the groups were compared by one-way ANOVA followed by Tukey’s multiple-comparison test. The results are representative of a single experiment repeated three times.

**Figure 5:**
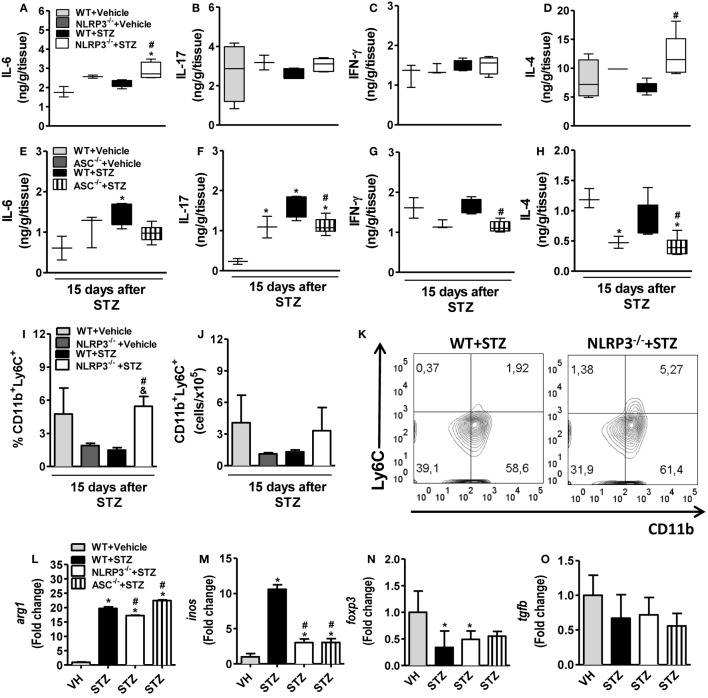
**NOD-like receptor family-pyrin domain containing 3 (NLRP3) deficiency potentiates IL-6 and IL-4 production and myeloid-derived suppressor cell (MDSC) expansion in pancreatic lymph nodes (PLNs) in type 1 diabetes**. Pancreatic tissue from vehicle-injected wild-type (WT) (light gray bars), NLRP3^−/−^, or ASC^−/−^ (dark gray bars) and streptozotocin (STZ)-administered WT (black bars), NLRP3^−/−^ (white bars), or ASC^−/−^ (striped bars) mice were harvested 15 days after the initiation of STZ or vehicle administration. The concentrations of IL-6 **(A,E)**, IL-17 **(B,F)**, IFN-γ **(C,G)**, or IL-4 **(D,H)** were determined in the pancreatic tissue by an ELISA assay. The percentage and absolute numbers of MDSC cells in the PLNs were determined by flow cytometry **(I,J)**. Monocytic MDSC percentages (CD11b^+^ Ly6C^+^) in the PLNs are shown in representative dot plots **(K)**. Relative gene expressions of arginase-1 **(L)**, iNOS **(M)**, Foxp3 **(N)**, and TGF-β **(O)** were measured by RT-PCR in the pancreatic tissue. The results are expressed as the mean ± SEM (*n* = 12 in the vehicle-injected WT group; *n* = 24 in the STZ-administered WT group; *n* = 12 in the vehicle-injected NLRP3^−/−^ group; and *n* = 24 in the STZ-administered NLRP3^−/−^ group). *p ≤ 0.05 compared to the vehicle-injected WT group, ^#^*p* ≤ 0.05 compared to the STZ-administered WT group, or ^&^*p* ≤ 0.05 compared to the vehicle-injected NLRP3^−/−^ group. Significant differences between the groups were compared by one-way ANOVA followed by Tukey’s multiple-comparison test. The results are representative of a single experiment repeated three times.

The percentage and absolute numbers of CD4^+^ T cells and CD8^+^ T cells were unaltered in the PLNs of diabetic WT mice compared to vehicle-treated mice (Figures 4A–D). However, the percentage of CD4^+^ T cells, but not CD8^+^ T cells, were significantly reduced in the PLNs of NLRP3^−/−^ mice compared to diabetic WT mice (Figures 4A,C). In addition, the CD4^+^IL-17^+^ cell frequency and absolute numbers in the PLNs from NLRP3^−/−^mice were significantly decreased compared to diabetic WT mice (Figures 4E,F,M). Similarly, the frequency and absolute numbers of CD8^+^IL-17^+^ cells were significantly reduced in the PLNs of these mice (Figures 4G,H). Despite a trend toward reduction, there was no significant difference between the percentage and absolute numbers of CD4^+^IFN-γ^+^ and CD8^+^IFN-γ^+^ cells in the PLNs of NLRP3^−/−^mice and diabetic WT mice (Figures 4I–L). In parallel, NLRP3 deficiency significantly increased IL-6 and IL-4 levels (Figures 5A,D) without alterations in the IL-17 and IFN-γ levels (Figures 5B,C) in the pancreatic tissue after STZ treatment. Surprisingly, ASC-deficient mice had significantly decreased IL-17, IFN-γ, and IL-4 (Figures 5F–H), but the IL-6 levels remained unaltered (Figure 5E). Our next step was to identify whether the resistance observed in NLRP3^−/−^ mice could be attributed to the induction of tolerogenic cells in the myeloid or lymphoid compartment. Importantly, NLRP3^−/−^ mice had a normalization of frequency and absolute numbers of MDSCs in the PLNs compared to diabetic WT mice (Figures 5I–K). On the other hand, the arginase-1 and iNOS gene expressions decreased in the pancreatic tissue of NLRP3^−/−^ mice compared to diabetic WT mice (Figures 5L,M), while Foxp3 and TGF-β gene expression was not altered (Figures 5N,O). Overall, our results showed that NLRP3 activation promotes IL-1β production, which in turn triggers Th17/Tc17 induction and dampens MDSC expansion in STZ-induced T1D.

### Mitochondrial DNA Triggers Caspase-1-Dependent IL-1β Production by Macrophages

**Figure 6:**
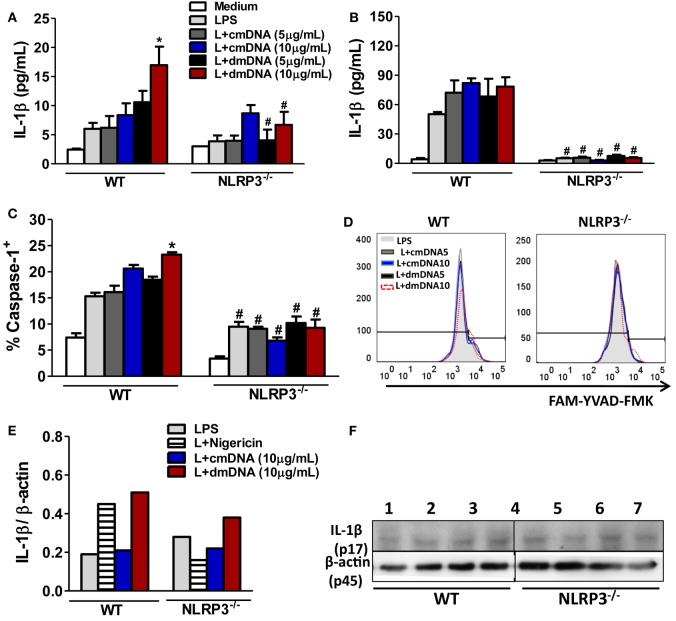
**NOD-like receptor family-pyrin domain containing 3 (NLRP3) activation by mitochondrial DNA (mDNA) from diabetic mice induces IL-1β expression and caspase-1 activation in macrophages**. Bone marrow-derived macrophages (BMDMs) were primed without (medium alone) or with LPS for 4 h (0.5 µg/mL). After that, they were incubated with nigericin at 20 µM for more 30 min or were incubated with mDNA from control mice (cmDNA) or from diabetic mice (dmDNA) at concentrations of 5 or 10 µg/mL for more 2 h. IL-1β **(A)** and IL-1α **(B)** concentrations in supernatants of BMDMs were measured by an ELISA assay. Caspase-1 activation in macrophages was detected with a fluorescent cell-permeable probe that binds to activated caspase-1 (FLICA) **(C,D)**. Cleaved IL-1β p17 subunit was detected by Western blotting. Bar graph represents the bands quantified by densitometric analysis **(E)**. BMDMs from wild-type (WT) (left panel) or NLRP3^−/−^ mice (right panel) were stimulated with LPS (L) only (1,5), or with LPS plus nigericin (2,6), plus cmDNA (3,7) or plus dmDNA (4,8) in the concentration of 10 µg/mL **(F)**. *p ≤ 0.05 compared to LPS-stimulated BMDMs or ^#^*p* ≤ 0.05 NLRP3^−/−^ vs. WT BMDMs after respective treatments. Significant differences between the groups were compared by one-way ANOVA followed by Tukey’s multiple-comparison test. Four biological replicates per group were used in each *in vitro* experiment. The results are representative of a single experiment repeated three times.

Later, we determined the effect of mDNA from non-diabetic mice (cmDNA) or from diabetic mice (dmDNA) on NLRP3 inflammasome activation. To examine whether the inflammasome is activated by mDNA, bone marrow-derived macrophages (BMDMs) were exposed to different concentrations of mDNA after priming with LPS to allow expression of pro-IL-1β. Consistently, IL-1β production was significantly increased in BMDMs from WT mice after stimulation with dmDNA (10 µg/mL) when compared with cells stimulated only with LPS. However, this response was reduced in BMDMs from NLRP3^−/−^ mice. On the other hand, cmDNA stimulation (5 or 10 µg/mL) after LPS priming did not induce IL-1β production (Figure 6A). Conversely, cmDNA or dmDNA stimulation (5 or 10 µg/mL) did not change IL-1α production in BMDMs from WT mice when compared to LPS-stimulated cells. Nevertheless, BMDMs from NLRP3^−/−^ mice already presented a significant IL-1α reduction when compared to BMDMs from WT mice, independent of the stimulus that was used (Figure 6B).

Because active caspase-1 is crucial for the cleavage of pro-IL-1β to its mature and biologically active form, we determined if dmDNA is able to trigger the activation of caspase-1. The activation of caspase-1 was demonstrated by using the FAMYVAD-FMK fluorescent inhibitor (FLICA), which binds covalently to active caspase-1. Notably, the dmDNA stimulation at a concentration of 10 µg/mL induced a significant increase in the percentage of caspase-1-positive BMDMs compared BMDMs incubated only with LPS, and this effect was inhibited in the BMDMs of NLRP3^−/−^ mice. However, cmDNA stimulation at a concentration of 5 or 10 µg/mL did not promote a significant increase in caspase-1-positive BMDMs compared to the LPS stimulus alone (Figures 6C,D). Because nigericin is a potent activator of the NLRP3 inflammasome, we used this compound as a positive control in the Western blot assays. Of fact, we detected an increase of active IL-1β (p17) expression in the supernatant of BMDMs from WT mice exposed to nigericin after LPS priming, which was inhibited in BMDMs from NLRP3^−/−^ mice. Similarly, the immunoblot analysis showed that the active form of IL-1β was being produced in response to dmDNA at a concentration of 10 µg/mL in the supernatant of the BMDMs from WT mice, but this effect was inhibited in BMDMs from NLRP3^−/−^ mice, after priming the cells with LPS. Of interesting manner, the expression of active form of IL-1β was similar by BMDMs from WT mice or NLRP3^−/−^ mice in response to cmDNA at the same concentration (Figures 6E,F). These results suggest that the NLRP3 inflammasome senses mitochondrial DNA from diabetic mice in macrophages and causes caspase-1-dependent IL-1β production.

### Mitochondrial DNA from Diabetic Mice Precipitates STZ-Induced T1D Onset

**Figure 7:**
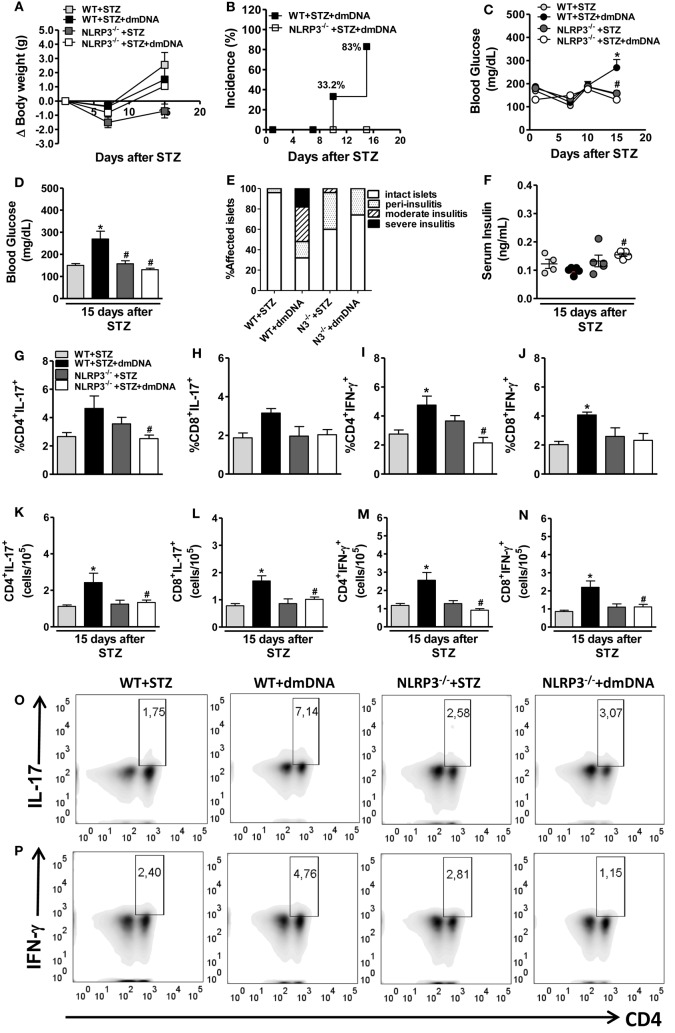
**NOD-like receptor family-pyrin domain containing 3 (NLRP3) activation by mitochondrial DNA (mDNA) from diabetic mice contributes to Th17/Tc17/Th1/Tc1 response and leads to type 1 diabetes onset**. Body weight variation **(A)**, cumulative disease incidence **(B)**, and time course of glycemia was monitored, or blood glucose levels were determined after 15 days of streptozotocin (STZ) **(C,D)** in wild-type (WT) mice treated only with four doses of STZ (light gray bars) plus mDNA from diabetic mice (dmDNA) (black bars) or NLRP3^−/−^ mice treated only with four doses of STZ (dark gray bars) plus dmDNA (white bars). These clinical parameters were monitored 1, 7, 10, and 15 days after the initial STZ treatment. The insulitis score was evaluated using a semiquantitative scale **(E)**. The serum insulin concentrations were determined at day 15 after the initiation of STZ **(F)**. The percentage and absolute numbers of CD4^+^IL-17^+^ **(G,K)**, CD8^+^IL-17^+^ **(H,L)**, CD4^+^IFN-γ^+^ **(I,M)**, and CD8^+^IFN-γ^+^ **(J,N)** cells in the pancreatic lymph nodes (PLNs) was determined by flow cytometry. Representative dot plots of the Th17 (CD4^+^IL-17^+^) and Th1 (CD4^+^IFN-γ^+^) percentages in the PLNs, respectively **(O,P)**. Intracellular cytokine levels were detected after stimulation with PMA plus ionomycin. The gate was set on CD3-positive lymphocytes. The results are expressed as the mean ± SEM (*n* = 12 in the WT group administered only with STZ; *n* = 18 in the WT group administered with STZ plus mDNA; *n* = 12 in the NLRP3^−/−^ group administered only with STZ; and *n* = 18 NLRP3^−/−^ group administered with STZ plus mDNA). *p ≤ 0.05 compared to the WT group treated only with STZ, ^#^*p* ≤ 0.05 compared to the WT group treated with STZ plus mDNA. Significant differences between the groups were compared by one-way ANOVA followed by Tukey’s multiple-comparison test. The results are representative of a single experiment repeated three times.

The administration of five doses of STZ (40 mg/kg) induces T1D as described before. However, the administration of only four doses does not have this effect (Figures 7A–D). This result suggests that four doses of STZ do not induce T1D because the doses are not sufficient to produce robust β-cell damage. Therefore, we tested whether four sub-diabetogenic doses of STZ would cause T1D if they were administered with mDNA from diabetic mice (three doses each at 5 µg i.p.) to C57BL/6 mice on day 1 before and days 6 and 9 after STZ administration. Our findings showed that dmDNA administration predisposes animals to T1D onset, which was confirmed by 83% disease incidence (Figure 7B) and increased blood glucose levels after four sub-diabetogenic doses of STZ (Figures 7C,D). Nevertheless, dmDNA administration did not affect body weight loss compared with mice only given four doses of STZ (Figure 7A). The dmDNA effects in the disease incidence and hyperglycemia were abrogated in NLRP3^−/−^ mice, since we observed a significant reduction of glycemic levels in these mice compared to diabetic WT mice after four doses of STZ (Figures 7B–D). As shown in Figure 7E, the group treated only with STZ had a striking proportion of insulitis-free islets (96%) and some infiltrated areas with peri-insulitis (4%). However, more infiltrated areas with peri-insulitis (16%), moderate insulitis (34%), and severe insulitis (18%) were observed in mice treated with four sub-diabetogenic doses of STZ and dmDNA. On the other hand, the pancreatic islets of NLRP3^−/−^ mice revealed more insulitis-free islets (74%) and no moderate or severe insulitis after the same treatments. Similarly, serum insulin levels further decreased in WT mice, but were normalized in NLRP3^−/−^ mice treated with dmDNA after STZ (Figure 7F). Thus, these data indicate that NLRP3 activation mediated by mDNA from diabetic mice is required for the pancreatic islet inflammation involved in insulin-producing β cell damage and T1D development.

### Mitochondrial DNA Induces Pathogenic Lymphocyte Response and Dampens Mast Cell and MDSC Expansion in STZ-Induced T1D

Considering dmDNA promotes IL-1β production mediated by NLRP3 and the established role of IL-1β in Th17 differentiation (21, 22), we examined whether NLRP3 is involved in Th17 and Tc17 induction in mice treated with mDNA after sub-diabetogenic doses of STZ. The dmDNA administration plus STZ was able to promote a significant increase of CD4^+^IL-17^+^ and CD8^+^IL-17^+^ absolute numbers in the PLNs of WT mice (Figures 7K,L). Importantly, the frequency and absolute numbers of CD4^+^IL-17^+^ cells were significantly reduced in the PLNs of NLRP3^−/−^ mice compared to diabetic WT mice treated with STZ and dmDNA (Figures 7G,K,O). Although there were no differences in the frequency, the absolute number of CD8^+^IL-17^+^ cells was significantly decreased in the PLNs of NLRP3^−/−^ mice compared to diabetic WT mice after the same treatments (Figures 7H,L). Another important observation is the increased frequency and absolute numbers of CD4^+^IFN-γ^+^ and CD8^+^IFN-γ^+^ cells in the PLNs of mice treated with mDNA after sub-diabetogenic doses of STZ (Figures 7I,J,M,N). Of note, the frequency and absolute numbers of CD4^+^IFN-γ^+^ cells were significantly decreased in the PLNs of NLRP3^−/−^ mice compared to diabetic WT mice treated with STZ and dmDNA (Figures 7I,M,P). However, only the absolute numbers of CD8^+^IFN-γ^+^ cells, but not the percentage, were significantly decreased in the PLNs of NLRP3^−/−^ mice compared to diabetic WT mice (Figures 7J,N).

Analysis of circulating mDNA genes, such as NADH dehydrogenase subunit 6 (NADH), cytochrome *b* (Cyt B), and cytochrome *c* (Cyt C), demonstrated a significant increase in gene expression of Cyt B and Cyt C, but not NADH, 15 days after STZ in diabetic mice compared to vehicle-treated mice (Figures S2A–C in Supplementary Material). Interestingly, we observed that NLRP3^−/−^ mice treated with STZ and dmDNA exhibited a significant increase in the percentage and absolute numbers of mast cells (Figures S2D,G in Supplementary Material), but not M2 macrophages (Figures 2E,H in Supplementary Material), compared to WT and NLRP3^−/−^ mice after only STZ doses. In addition, NLRP3^−/−^ mice had a trend to increase the percentage and absolute number of monocytic MDSCs compared to WT mice treated with STZ and dmDNA (Figures S2F,I in Supplementary Material). A coadministration of STZ and dmDNA also significantly increased IL-1β levels in the pancreatic tissue of WT mice, but significantly decreased IL-1β levels in NLRP3-deficient mice (Figure S2J in Supplementary Material). Conversely, the NLRP3 deficiency caused a significant increase in IL-6 levels (Figure S2K in Supplementary Material) without affecting the IL-17, IL-23, IFN-γ, and IL-10 levels (Figures S2L–O in Supplementary Material) in the pancreatic tissue after dmDNA and STZ administration. Taken together, our results showed that NLRP3 activation depended on mDNA from diabetic mice for the induction of Th17/Tc17/Th1/Tc1 responses and the suppression of mast cells and MDSCs in STZ-induced T1D.

## Discussion

Type 1 diabetes is one of the most prevalent autoimmune diseases in the world. It affects approximately 10–20 million people and develops most frequently in childhood but also can develop in adulthood. Similar to other autoimmune disorders, the etiology of diabetes remains unclear, but it is known that the risk of developing the disease is determined by genetic and environmental factors, including viral infections, food, vaccination, toxins, and stress (23, 24). A strong association between NLRP3 polymorphisms and a greater predisposition to the disease has been reported in diabetic patients (19).

The NLRP3 inflammasome is a molecular platform required for the proteolytic cleavage of caspase-1 and is activated by endogenous and exogenous stimuli, including uric acid crystals and silica, bacterial toxins, β-amyloid particles, and ATP (12–14). After activation, NLRP3 oligomerization and interaction with the adapter molecule ASC resulted in activation of caspase-1 and expression of active forms of IL-1β and IL-18. Our results showed a correlation between increased NLRP3, ASC, and pro-IL-1β gene expression in the PLNs, as well as IL-1β, but not IL-18 protein expression at day 7 in the pancreatic tissue of STZ-induced diabetic mice. In addition, pancreatic IL-1β expression remained elevated at day 15 through a mechanism dependent on NLRP3 inflammasome activation. IL-18 expression, after 15 days of STZ-induced T1D, was not dependent on NLRP3 inflammasome activation. In parallel, we observed an elevated percentage of caspase-1-expressing macrophages in the PLNs of diabetic mice, which was reduced in mice lacking NLRP3. In addition, deficiency of IL-1R and NLRP3 in mice triggered resistance to T1D development. This protection observed in IL-1R^−/−^ mice was associated with smaller IL-17 production in the pancreatic tissue during T1D. NLRP3 deficiency in NOD mice also protected against T1D through inhibition of chemokines CCL5 and CXCL10 in the pancreatic islets (20). Taken together, these results indicate that NLRP3-dependent IL-1β production accounts for T1D onset in the STZ-induced experimental model.

NLRP3 receptor activation plays a crucial role in the induction of inflammatory responses and in the subsequent polarization of the adaptive immune response. In terms of cellular immunity, T CD4 lymphocytes are related to Th1, Th2, Treg, Th9, Th22, and Th17 according to their profile of cytokine expression and transcription factors (25). The differentiation of these cell subtypes is induced by a differential pattern of anti- or pro-inflammatory cytokines produced by macrophages and dendritic cells (26). It has been reported that IL-18 and IL-1β play an important role in driving Th1 and Th17 cellular responses, respectively (27). Initial studies supported a crucial role for IFN-γ-secreting Th1 cells in T1D pathogenesis (28, 29). However, this notion was altered by the discovery that genetic absence neither IFN-γ nor its receptor protect against T1D in NOD mice (30, 31). Notably, Emamaullee et al. provided strong evidence about the pathogenic role of Th17 in T1D by treating animals with either a neutralizing anti-IL-17 antibody or recombinant IL-25 (32). The deficiency of IL-1R, as well as NLRP3 in mice protected against T1D development and was associated with reduced Th17/Tc17/Th1 populations in the PLNs. Previous studies have also reported that deficiency of IL-1R or NLRP3 results in a lower production of IL-17 and causes resistance to EAE (16, 33). Similarly, another study reported that IL-1Ra-deficient mice spontaneously develop arthritis due to the high expression of IL-17 caused by increased signaling of IL-1 (34).

Mechanisms involving IL-1β-induced Th17 differentiation have been reported. TGF-β induces ROR-γt expression in naïve T cells and triggers IL-23R and IL-1R expression, which makes these cells receptive to IL-23 and IL-1β (35). *In vitro* studies have shown that IL-1β induces the expression of IRF-4, which positively regulates IL-21-mediated STAT-3 and ROR-γt transcription factor expression (18, 36). Additionally, a role for IL-1β on Th17 phenotype induction has been attributed to alternative splicing of Foxp3 (37). However, there are no reports about the direct effect of IL-1β on the induction or expansion of Th1 lymphocytes. More recently, the ability of Th17 and Tc17 lymphocytes to be converted into Th1 lymphocytes in the presence of IL-12 in T1D was also observed (38, 39), demonstrating the considerable plasticity of these cellular subtypes. Therefore, it is possible that the reduction in the Th1 lymphocyte population in NLRP3^−/−^ mice is due to a defect in the differentiation of Th17/Tc17 lymphocytes and their subsequent conversion into Th1 lymphocytes. Taken together, our results demonstrate that NLRP3-dependent IL-1β expression appears to drive Th17/Tc17/Th1 differentiation under inflammatory conditions, such as T1D.

NLRP3 deficiency increased IL-6 and IL-4 protein expression in the pancreatic tissue after STZ administration. A study has shown that the activation of basophils and mast cells induces the secretion of IL-4 and delays the onset of T1D in NOD mice (40). Additionally, coculturing mast cells with MDSC leads to IL-6, IL-13, and TNF-α production, increasing their suppressor activity (41). MDSCs are increased in the blood of patients and experimental models of T1D, but these cells have a defect in their suppressor activity (42). Supporting these findings, we also observed a positive correlation between increased numbers of mast cells and MDSCs in the PLNs of NLRP3^−/−^ mice after STZ administration, which indicates a synergistic effect between these two cell subtypes in protection against T1D. More recently, IL-6 has been involved in the generation of both mouse and human MDSC cells (43, 44). Accordingly, we demonstrated that mast cells play a regulatory role through IL-6-dependent mechanisms during T1D (45). Based on this evidence, we suggest that the increased MDSC population in NLRP3^−/−^ mice is due to elevated IL-6 expression, which in turn inhibits the inflammatory response in the pancreatic islets and prevents the onset of T1D.

Danger-associated molecular patterns are usually found in different compartments within cells and are often modified by proteolytic and oxidative processes associated with cellular injury mechanisms (46). Most DAMPs are released or secreted and exert their biological activity through the activation of TLR and NLR receptors (47). In particular, mitochondrial DAMPs, including mDNA and formylated peptides, stimulate neutrophils *via* TLR9 and FPR-1, respectively, after being released into the extracellular space (48). In humans, the presence of mDNA is detected in the synovial fluid of patients with arthritis, and intra-articular injection of mDNA induces arthritis mediated by the recruitment of macrophages and monocytes. Researchers concluded that oxidatively damaged DNA bases are major contributors to arthritis development (49). Another study also revealed that neutrophil mitochondria guide oxidized mDNA in the steady state into lysosomes for degradation. On the other hand, blood neutrophils from patients with systemic lupus erythematosus (SLE) patients have mitochondrial retention of oxidized nucleoids, indicating that a defect in degradation of neutrophil oxidized mDNA might contribute to SLE pathogenesis (50).

It is known that excessive oxidative damage to DNA impairs the normal repair mechanisms and induces apoptosis (51, 52).

Considering that alterations in DNA induced by oxidative stress contribute to diabetes progression (53), we addressed the *in vitro* and *in vivo* effects of mDNA released in response to pancreatic damage in T1D in the activation of NLRP3 inflammasome in macrophages. Macrophages primed with LPS and stimulated with mDNA from diabetic mice exhibited increased IL-1β production, caspase-1 expression and cleavage of pro-IL-1β in active IL-1β *in vitro*. In addition, coadministration of mDNA plus four doses of STZ-induced pancreatic islet inflammation and led to T1D, which was abrogated in NLRP3-deficient mice. mDNA plasma levels are significantly elevated in diabetic patients compared with healthy controls (54). Likewise, we also detected increased expression of mDNA-related genes in the serum of diabetic mice. It is plausible that tissue necrosis resulting from beta-cell death leads to extracellular mDNA release. However, it is still puzzling the fact that only mDNA from diabetic mice activates the NLRP3 inflammasome. We speculate that beta-cell death and DAMP release, such as mDNA, occur during the initial phase of diabetes (prediabetic phase). In addition, oxidative stress that contributes to beta-cell death may induce mDNA oxidation, which turns into an immunogenic molecule. In this context, it has been reported that ATP induces mitochondrial dysfunction, apoptosis, and oxidized mDNA release into the cytosol, which activates the NLRP3 inflammasome (12). Thus, it seems likely that the presence of extracellular mDNA exacerbates inflammation by stimulating IL-1β production *via* NLRP3 activation, thereby causing massive β-cell destruction and accelerating T1D onset in this experimental model.

In summary, we conclude that NLRP3 inflammasome activation mediated by mitochondrial DNA from diabetic mice promotes caspase-1 activation and IL-1β production by macrophages, which drives pathogenic Th17/Tc17/Th1 responses and negatively modulates the tolerogenic responses mediated by MDSC and mast cells in the PLNs, and leads to the development of T1D. Thus, alternative therapies using nucleases or drugs that cause extracellular mDNA degradation should be explored in human T1D.

## Animals and Methods

### Animals

This research project was approved by the Animal Research Ethics Committee of the Ribeirao Preto Medical School, University of São Paulo (no. 001/2008). Male NLRP3^−/−^, IL-1R^−/−^, and ASC^−/−^ mice generated on the C57BL/6 background (8–12 weeks old) were obtained from the Isogenic Breeding Unit at Ribeirao Preto Medical School, University of São Paulo, Ribeirao Preto, Brazil. Female NOD/LtJ mice (8–20 weeks old) were obtained from the Jackson Laboratory and housed in the animal facility of the Department of Biochemistry and Immunology, Ribeirao Preto Medical School, at 23–25°C with free access to water and food.

### Induction of Diabetes by MLD-STZ

The mice were given daily intraperitoneal injections of 40 mg/kg of streptozotocin (Sigma-Aldrich,) dissolved in 0.1 M sodium citrate (pH 4.5) for five consecutive days. Blood glucose levels, body weight, and diabetes incidence were monitored weekly. Mice were defined as diabetic when glucose levels were ≥230 mg/dL after two consecutive determinations under non-fasting conditions.

### Flow Cytometry Analysis of Intracellular and Extracellular Markers

Flow cytometry analysis was performed on samples with 1 × 10^6^ cells/tube in 100 µL of PBS. First, cell suspensions were incubated with 5% normal rabbit serum for 30 min to block non-specific binding. Next, antibodies against CD3, CD4, CD8, CD25, CD117, FcϵRI, CD11b, Ly6C, CD206, TLR2, and their control isotypes (BD Pharmingen, San Diego, CA, USA) were added and incubated for 30 min in the dark. IL-17 and IFN-γ production was evaluated after *in vitro* reactivation with PMA (25 ng/mL) and ionomycin (1 mg/mL, Sigma-Aldrich) together with 10 mg/mL monensin (Sigma-Aldrich) as previously described (55). The cells were analyzed using a FACS Canto flow cytometer, and the data were analyzed using FlowJo (Tree Star) software.

### Detection of Cytokine Levels in Pancreatic Tissue

Pancreatic fragments (tail portions) were removed, weighed, and placed in a tube containing 700 µL of Complete Protease Inhibitor Cocktail (Roche Diagnostics, Abbott Park, IL, USA). The tissue was homogenized using a Polytron homogenizer (Thermo Fisher Scientific, Waltham, MA, USA) and IL-1∝, IL-1β, IL-18, IL-6, IL-17, IL-23, TNF-α, IFN-γ, IL-10, and IL-4 levels were detected by ELISA using colorimetric kits according to the manufacturer’s instructions (R&D Systems). The results were expressed as the mean nanograms ± SEM per gram/tissue (pancreatic tissue) or picograms per milliliter (culture supernatant).

### Quantification of Serum Insulin Levels

Serum samples were collected 15 days after MLD-STZ administration of non-fasting mice. The insulin concentration was determined using the Mouse Ultrasensitive Insulin ELISA kit (Alpco Diagnostics) according to the manufacturer’s instructions.

### Histological and Immunohistochemistry Analysis

Pancreatic fragments (head portion) were removed, fixed in 10% buffered formalin, and embedded in paraffin. Then, 4–5 µm sections were stained with hematoxylin and eosin (Merck, Whitehouse Station, NJ, USA). Immunohistochemistry reactions were performed as previously described (55). The degree of insulitis was evaluated using a semiquantitative scale: 0, intact islet; 1, peri-insulitis; 2, moderate insulitis (<50% of the islets infiltrated); and 3, severe insulitis (>50% of the islets infiltrated).

### Culture of BMDMs

The BMDMs from WT and NLRP3^−/−^ mice were differentiated as previously described (56). Briefly, total bone marrow cells were cultured for 7 days in RPMI 1640 medium (Sigma-Aldrich) supplemented with 10% fetal bovine serum (FBS) (Life Technologies, Molecular Probes, Carlsbad, CA, USA) and 30% L-929 cell-conditioned media at 37°C and 5% CO_2_. The cells (0.5 × 10^6^/well) were stimulated with nigericin (20 µM) for 30 min (Sigma-Aldrich), and then mitochondrial DNA from non-diabetic or control mice (cmDNA) or diabetic mice (dmDNA) at the concentration of 5 or 10 µg/mL for 2 h. Prior to stimulation, BMDMs were prestimulated for 4 h with LPS (0.5 µg/mL) (InvivoGen).

### Western Blotting

Fifty micrograms of extracted proteins were loaded directly into sodium dodecyl sulfate (SDS) sample buffer for 10% SDS-polyacrylamide gel electrophoresis. After transferring the samples onto a nitrocellulose membrane (Trans-Blot Transfer Medium; Bio-Rad, Hercules, CA, USA), the membranes were blocked with 5% milk in Tris buffer solution containing 0.1% Tween 20 for 1 h and then incubated with antibodies against IL-1β (Santa Cruz) or NLRP3 (R&D Systems) overnight at 4°C. Next, the cells were incubated with an IgG HRP-conjugated secondary Ab (Cell Signaling) for 1 h at room temperature. After the membranes were rinsed, the immunocomplexes were developed using an enhanced peroxidase/luminol chemiluminescence reaction (ECL Western blotting detection reagents; Pierce Biotechnology) and exposed to X-ray film with autoradiography (Carestream Health). The bands were quantified densitometrically using ImageTool 2.0 software (University of Texas), and the results were expressed as arbitrary units.

### Active Caspase-1 Staining

Active caspase-1 was detected using the caspase-1 fluorochrome inhibitor of caspases (FLICA) kit (Immunochemistry Technologies) according to the manufacturer’s instructions. Briefly, macrophages or PLN cells were adjusted to a volume of 0.5 × 10^6^/tube or 1-2 × 10^6^/tube, respectively. Later, the cells were stained for F4/80 and FLICA for 30 min at 37°C. The cells were then washed two times with PBS containing 10% FBS and analyzed directly with flow cytometry on a FACS Canto flow cytometer.

### Mitochondrial DNA Isolation

Pancreata from diabetic and non-diabetic mice (control) were used in protocols for isolating the mitochondria. Briefly, the pancreatic tissue was cut in pieces and added to 50 mL of medium (Hepes 10 mM, saccharose 250 mM e EGTA 1 mM) at pH 7.2 and homogenized for 15 s. Later, the pancreatic tissue was centrifuged at 600 *g* for 5 min, and the supernatant was collected and centrifuged at 2,000 *g* for 10 min. The pellet containing the isolated mitochondria was recovered. The mitochondria were sonicated at an amplitude of 100% (10 sonicagens of 30 s with 30 s intervals). Then, the suspension of lysed mitochondria was centrifuged at 12,000 *g* for 10 min at 4°C followed by centrifugation at 100,000 *g* at 4°C for 30 min. The supernatant from this centrifugation was used for DNA extraction with the phenol–chloroform–isoamyl alcohol mixture (Sigma-Aldrich). Finally, DNA quantitation was determined with a Nanodrop 2000 (Thermo Technologies).

### Mitochondrial DNA Quantification

Circulating DNA was extracted and purified using the QIAamp DNA Blood Mini Kit (Qiagen, Germantown, MD, USA). Isolated DNA from mice was amplified and quantified using real-time (RT)-PCR. The primers (Invitrogen, Grand Island, New York, NY, USA) that were used to amplify mDNA were cytochrome *b* (Cyt *b*) (forward 5-ACCTCAAAGCAACGAAGCCT-3′ and reverse 5′-GGTTGGCCTCCAATTCAGGT-3′), cytochrome *c* (Cyt *c*) (forward 5′-GACTTGCAACCCTACACGGAT-3′ and reverse 5′-CCGGTTAGACCACCAACTGT-3′), and NADH dehydrogenase subunit 6 (forward 5′-ATTCCACCCCCTCACGACTA-3′ and reverse 5′-TGTCGTTTTGGGTGAGAGCA-3′). The primer sequences have no homology with DNA found in any bacterial species published on BLAST. The RT-PCR results are presented as the inverse of cycle threshold (CT) for gene amplification as described (57).

### RNA Extraction and Quantitative RT-PCR

Total RNA was extracted from the PLNs or pancreatic tissue using Trizol (Life Technologies, Molecular Probes, Carlsbad, CA, USA) following the manufacturer’s instructions. cDNA was obtained using a High Capacity reverse transcription kit (Applied Biosystems, Foster City, CA, USA). Quantitative mRNA analysis by RT PCR was performed using the SYBR Green fluorescence system (Applied Biosystems). The following primers were used: β-actin: 5′-AACGAGCGGTTCCGATG-3′, reverse: 5′-GGATTCCATACCCAACAAGGA-3′, NLRP3 forward: 5′-GTGGATGGGTTTGCTGGGAT-3′, reverse: 5′-CCACACTCTACCTAGACGC-3′; IL-1β forward: 5′-TGACAGTGATGAGAATGACCTGTTC-3′, reverse: 5′-TTGGAAGCAGCCCTTCATCT-3′; arginase-1 forward: 5′-GTTCCCAGATGTACCAGGATTC-3′, reverse: 5′-CGATGTCTTTGGCAGATATGC-3′; iNOS forward: 5′-CGTGAGTGGAGTCATACTGGAA-3′, reverse: 5′-CGAAACGCTTCACTTCCAA-3′; TGF-β forward: 5′-TGAACCAAGGAGACGGAATACA-3′, reverse: 5′-GGAGTTTGTTATCTTTGCTGTCACA-3′; Foxp3 forward: 5′-ACAACCTGAGCCTGCACAAGT-3′, reverse: 5′-GCCCACCTTTTCTTGGTTTTG-3′. Specific mRNA expression levels were normalized relative to β-actin mRNA levels using the comparative 2ΔΔ*C*_t_ method.

### Statistical Analysis

The data are expressed as the mean ± SEM. The differences observed among the several experimental groups were performed by applying one-way ANOVA followed by the parametric Tukey’s test for comparing multiple groups or by Student’s *t*-test for comparing two groups. The incidence curve was analyzed by Mantel–Cox log-rank test. All analyses were performed using Prism 5.0 software (GraphPad Software). Statistical significance was set at *p* < 0.05.

## Author Contributions

DC carried out the experimental design, performed experiments, analyzed the results, and wrote the manuscript; FC, FR, and JY contributed to the analysis and interpretation of data and helped with *in vivo* experiments; CP and GO participated in the acquisition and interpretation of data and helped with *in vitro* experiments; SR supported us with histology and imaging data. FC, RT, and NC edited the manuscript, provided scientific assistance, and revised it critically, and JS provided intellectual support in addition to directing and supervising the study.

## Conflict of Interest Statement

The authors declare that the research was conducted in the absence of any commercial or financial relationships that could be construed as a potential conflict of interest.

## Supplementary Material

Figure S1 **IL-1R deficiency reduces the Th17/Tc17/Th1 cell population in the pancreatic lymph nodes (PLNs) in type 1 diabetes**. The percentage and absolute numbers of CD4^+^IL-17^+^ **(A,D)**, CD8^+^IL-17^+^ **(B,E)**, and CD4^+^IFN-γ^+^ **(C,F)** cells in the PLNs were determined by flow cytometry. Intracellular cytokine levels were detected after stimulation with PMA plus ionomycin. The gate was set on CD3-positive lymphocytes. The concentrations of IL-6 **(G)**, IL-17 **(H)**, IFN-γ **(I)**, TNF-α **(J)**, IL-4 **(K)**, and IL-10 **(L)** were determined in the pancreatic tissue by an ELISA assay. The results are expressed as the mean ± SEM (*n* = 9 in the vehicle-injected wild-type (WT) group; *n* = 24 in the streptozotocin (STZ)-administered WT group; *n* = 9 in the vehicle-injected IL-1R^−/−^ group; and *n* = 24 in the STZ-administered IL-1R^−/−^ group). **p* ≤ 0.05 compared to the vehicle-injected WT group, ^#^*p* ≤ 0.05 compared to the STZ-administered WT group. Significant differences between the groups were compared by one-way ANOVA followed by Tukey’s multiple-comparison test. The results are representative of a single experiment repeated three times.

Figure S2 **NOD-like receptor family-pyrin domain containing 3 (NLRP3) activation by mitochondrial DNA (mDNA) from diabetic mice decreases the mast cell and myeloid-derived suppressor cell population in the pancreatic lymph nodes (PLNs)**. Relative quantification of circulating mDNA genes, NADH dehydrogenase subunit 6 (NADH), cytochrome *b* (Cyt *b*), and cytochrome *c* (Cyt *c*) measured by RT-PCR of vehicle-injected or streptozotocin (STZ)-administered mice **(A–C)**. The percentage and absolute numbers of CD117^+^FcϵRI^+^ **(D,G)**, CD11b^+^CD206^+^ **(E,H)**, and CD11b^+^Ly6C^+^ **(F,I)** in the PLNs were determined by flow cytometry. The concentrations of IL-1β **(J)**, IL-6 **(K)**, IL-17 **(L)**, IL-23 **(M)**, IFN-γ **(N)**, or IL-10 **(O)** were determined in the pancreatic tissue by an ELISA assay. The results are expressed as the mean ± SEM [*n* = 12 in the wild-type (WT) group administered only with STZ; *n* = 18 in the WT group administered with STZ plus mDNA; *n* = 12 in the NLRP3^−/−^ group administered only with STZ; and *n* = 18 NLRP3^−/−^ group administered with STZ plus mDNA]. **p* ≤ 0.05 compared to the vehicle-injected WT group or treated only with STZ, ^#^*p* ≤ 0.05 compared to the WT group or treated only with STZ plus mDNA, or ^&^*p* ≤ 0.05 compared to the NLRP3^−/−^ group administered only STZ. Significant differences between the groups were compared by one-way ANOVA followed by Tukey’s multiple-comparison test. The results are representative of a single experiment repeated three times.
