# Supplementary figures and images for: Mitochondrial DNA Activates the NLRP3 Inflammasome and Predisposes to Type 1 Diabetes in Murine Model
Source: Front Immunol. 2017 Feb 27;8:164. doi: 10.3389/fimmu.2017.00164 (PMC5326761; doi:10.3389/fimmu.2017.00164)

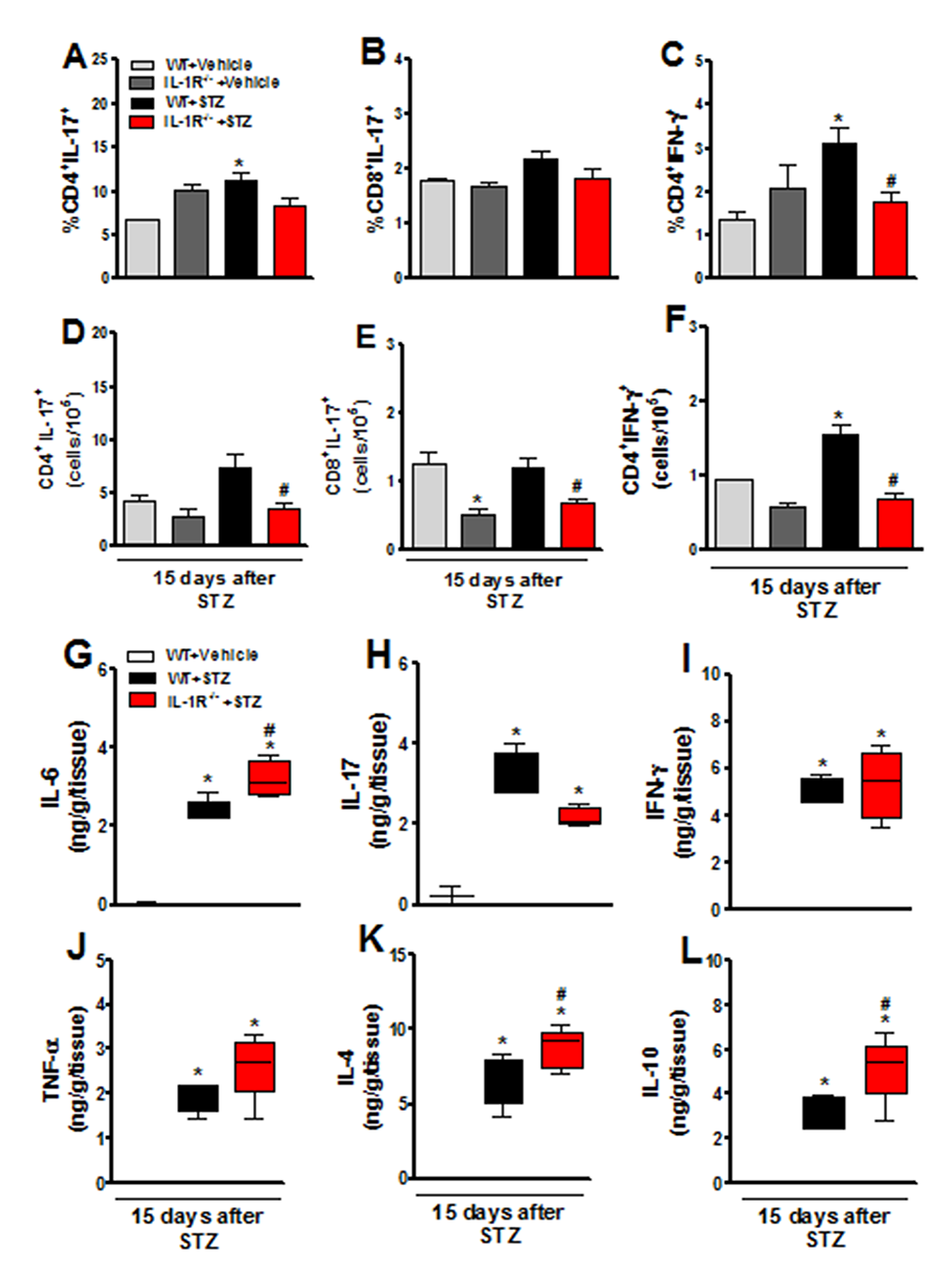

Supplement: Figure S1 — IL-1R deficiency reduces the Th17/Tc17/Th1 cell population in the pancreatic lymph nodes (PLNs) in type 1 diabetes. The percentage and absolute numbers of CD4+IL-17+ (A,D), CD8+IL-17+ (B,E), and CD4+IFN-γ+ (C,F) cells in the PLNs were determined by flow cytometry. Intracellular cytokine levels were detected after stimulation with PMA plus ionomycin. The gate was set on CD3-positive lymphocytes. The concentrations of IL-6 (G), IL-17 (H), IFN-γ (I), TNF-α (J), IL-4 (K), and IL-10 (L) were determined in the pancreatic tissue by an ELISA assay. The results are expressed as the mean ± SEM (n = 9 in the vehicle-injected wild-type (WT) group; n = 24 in the streptozotocin (STZ)-administered WT group; n = 9 in the vehicle-injected IL-1R−/− group; and n = 24 in the STZ-administered IL-1R−/− group). *p ≤ 0.05 compared to the vehicle-injected WT group, #p ≤ 0.05 compared to the STZ-administered WT group. Significant differences between the groups were compared by one-way ANOVA followed by Tukey’s multiple-comparison test. The results are representative of a single experiment repeated three times. [file image_1.tif]

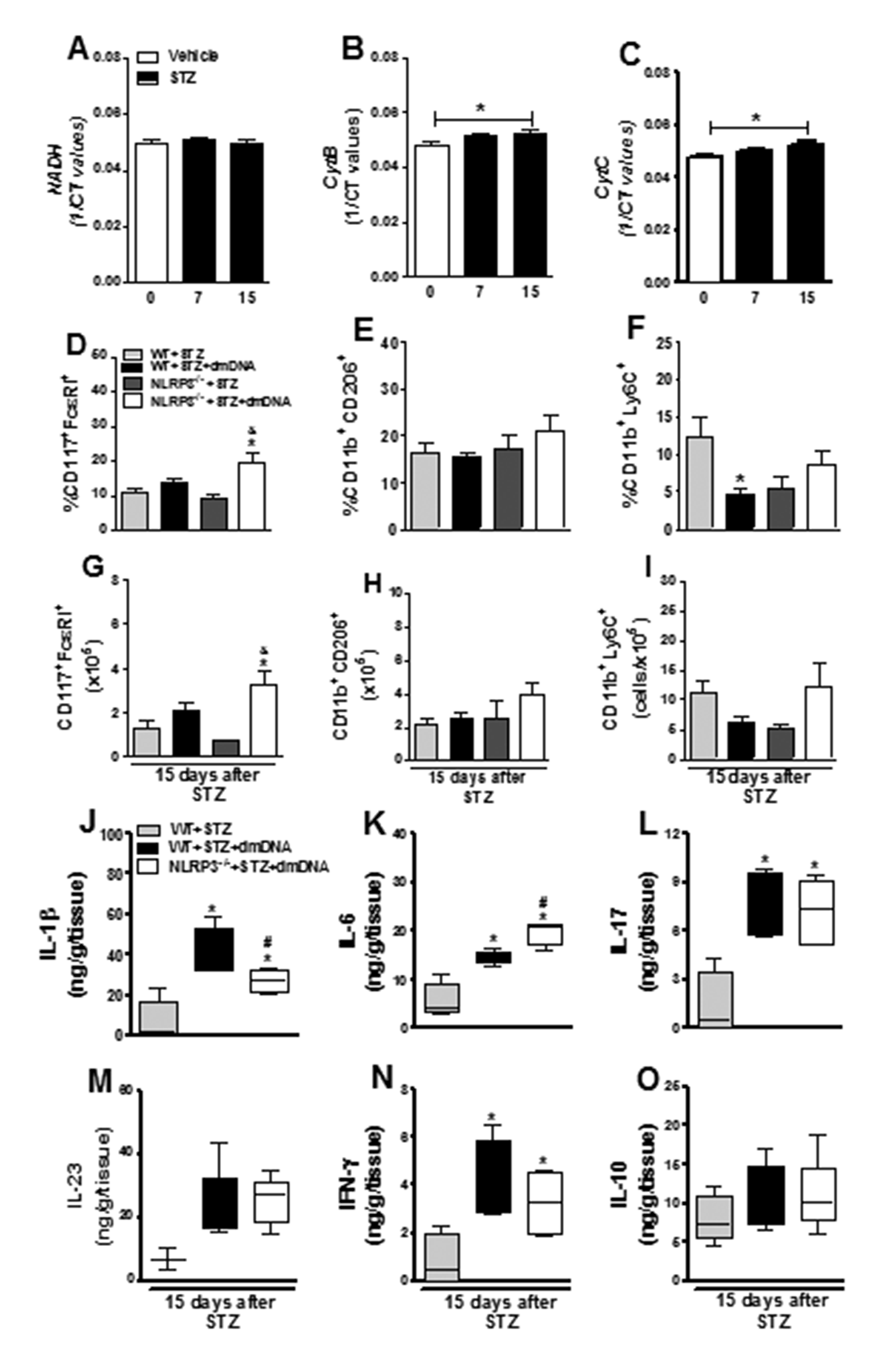

Supplement: Figure S2 — NOD-like receptor family-pyrin domain containing 3 (NLRP3) activation by mitochondrial DNA (mDNA) from diabetic mice decreases the mast cell and myeloid-derived suppressor cell population in the pancreatic lymph nodes (PLNs). Relative quantification of circulating mDNA genes, NADH dehydrogenase subunit 6 (NADH), cytochrome b (Cyt b), and cytochrome c (Cyt c) measured by RT-PCR of vehicle-injected or streptozotocin (STZ)-administered mice (A–C). The percentage and absolute numbers of CD117+FcϵRI+ (D,G), CD11b+CD206+ (E,H), and CD11b+Ly6C+ (F,I) in the PLNs were determined by flow cytometry. The concentrations of IL-1β (J), IL-6 (K), IL-17 (L), IL-23 (M), IFN-γ (N), or IL-10 (O) were determined in the pancreatic tissue by an ELISA assay. The results are expressed as the mean ± SEM [n = 12 in the wild-type (WT) group administered only with STZ; n = 18 in the WT group administered with STZ plus mDNA; n = 12 in the NLRP3−/− group administered only with STZ; and n = 18 NLRP3−/− group administered with STZ plus mDNA]. *p ≤ 0.05 compared to the vehicle-injected WT group or treated only with STZ, #p ≤ 0.05 compared to the WT group or treated only with STZ plus mDNA, or &p ≤ 0.05 compared to the NLRP3−/− group administered only STZ. Significant differences between the groups were compared by one-way ANOVA followed by Tukey’s multiple-comparison test. The results are representative of a single experiment repeated three times. [file image_2.tif]
